# 4-(Prop-2-yn­yl)-2*H*-1,4-benzo­thia­zin-3(4*H*)-one

**DOI:** 10.1107/S160053681400943X

**Published:** 2014-05-10

**Authors:** Nada Kheira Sebbar, Abdelfettah Zerzouf, El Mokhtar Essassi, Mohamed Saadi, Lahcen El Ammari

**Affiliations:** a Laboratoire de Chimie Organique Hétérocyclique URAC 21, Pôle de Compétence Pharmacochimie, Av. Ibn Battouta, BP 1014, Faculté des Sciences, Université Mohammed V-Agdal, Rabat, Morocco; b Laboratoire de Chimie Organique et Etudes Physicochimiques, ENS Takaddoum, Rabat, Morocco; c Laboratoire de Chimie du Solide Appliquée, Faculté des Sciences, Université Mohammed V-Agdal, Avenue Ibn Battouta, BP 1014, Rabat, Morocco

## Abstract

In the title compound, C_11_H_9_NOS, the six-membered heterocycle of the benzo­thia­zine fragment exhibits a screw-boat conformation. The benzene ring makes a dihedral angle of 79.4 (1)° with the mean plane through the prop-2-ynyl chain and the ring N atom. In the crystal, mol­ecules are linked by C—H⋯O inter­actions of the acetyl­enic C—H group towards the carbonyl O atom of a neighbouring mol­ecule, forming zigzag chains running along the *b*-axis direction.

## Related literature   

For general background to the synthesis of 1,4-benzo­thia­zines derivatives, see: Sebbar *et al.* (2014 ▶); Zerzouf *et al.* (2001 ▶). For the pharmacological activity of 1,4-benzo­thia­zine derivatives, see: Trapani *et al.* (1985 ▶); Yaltirik *et al.* (2001 ▶); Wammack *et al.* (2002 ▶). For puckering parameters, see: Cremer & Pople (1975 ▶).
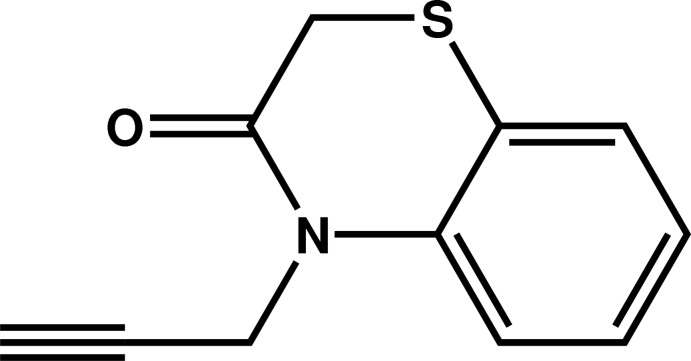


## Experimental   

### 

#### Crystal data  

C_11_H_9_NOS
*M* *_r_* = 203.25
Monoclinic, 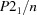

*a* = 9.005 (2) Å
*b* = 10.889 (3) Å
*c* = 10.341 (3) Å
β = 104.565 (7)°
*V* = 981.3 (4) Å^3^
*Z* = 4
Mo *K*α radiation
μ = 0.29 mm^−1^
*T* = 296 K
0.39 × 0.34 × 0.28 mm

#### Data collection  

Bruker X8 APEX diffractometer
Absorption correction: multi-scan (*SADABS*; Bruker, 2009 ▶) *T*_min_ = 0.692, *T*_max_ = 0.747
9586 measured reflections
2532 independent reflections
2242 reflections with *I* > 2σ(*I*)
*R*_int_ = 0.025

#### Refinement  

*R*[*F* ^2^ > 2σ(*F* ^2^)] = 0.033
*wR*(*F* ^2^) = 0.097
*S* = 1.05
2532 reflections
127 parameters
H-atom parameters constrained
Δρ_max_ = 0.29 e Å^−3^
Δρ_min_ = −0.26 e Å^−3^

### 

Data collection: *APEX2* (Bruker, 2009 ▶); cell refinement: *SAINT-Plus* (Bruker, 2009 ▶); data reduction: *SAINT-Plus*; program(s) used to solve structure: *SHELXS97* (Sheldrick, 2008 ▶); program(s) used to refine structure: *SHELXL97* (Sheldrick, 2008 ▶); molecular graphics: *ORTEP-3 for Windows* (Farrugia, 2012 ▶); software used to prepare material for publication: *PLATON* (Spek, 2009 ▶) and *publCIF* (Westrip, 2010 ▶).

## Supplementary Material

Crystal structure: contains datablock(s) I. DOI: 10.1107/S160053681400943X/im2453sup1.cif

Structure factors: contains datablock(s) I. DOI: 10.1107/S160053681400943X/im2453Isup2.hkl

Supporting information file. DOI: 10.1107/S160053681400943X/im2453Isup3.cml

CCDC reference: 999603

Additional supporting information:  crystallographic information; 3D view; checkCIF report

## Figures and Tables

**Table 1:** Hydrogen-bond geometry (Å, °)

*D*—H⋯*A*	*D*—H	H⋯*A*	*D*⋯*A*	*D*—H⋯*A*
C11—H11⋯O1^i^	0.93	2.32	3.1937 (19)	157

Symmetry code: (i) 
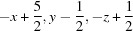
.

**Fig. 1.**
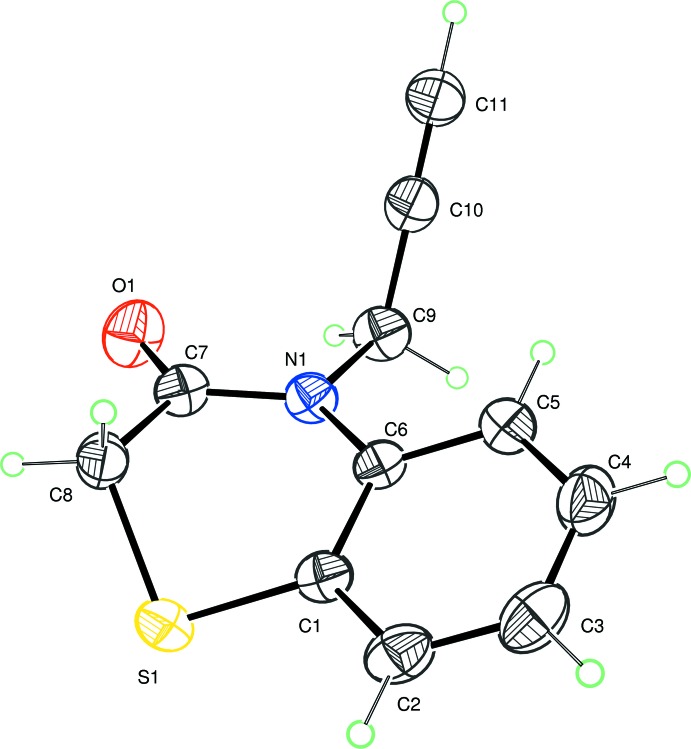
Molecular structure of the title compound with the atom-labelling scheme. Displacement ellipsoids are drawn at the 50% probability level. H atoms are represented as small circles.

**Fig. 2.**
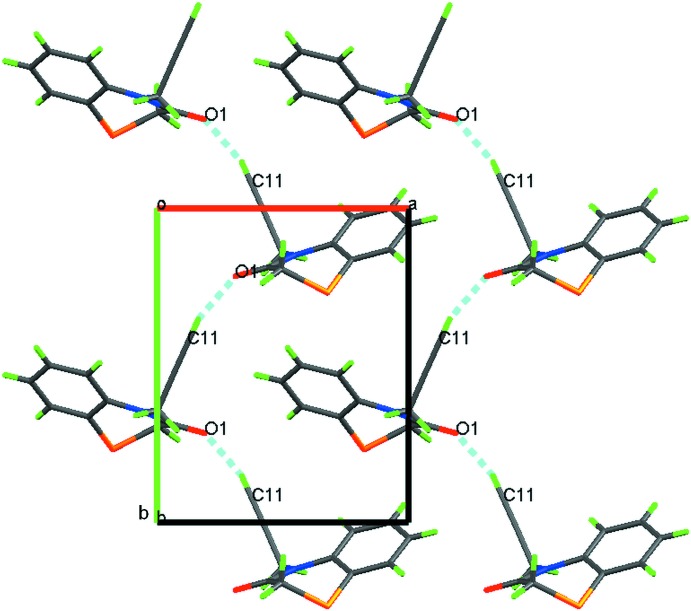
Projection of the structure along (0 0 1) of the title compound, showing molecules linked through C113–H11···O1 hydrogen bond (dashed lines).

## supplementary crystallographic information

### 1. Comment

1,4-Benzothiazine derivatives constitute an important class of heterocyclic compounds which possess a wide range of therapeutic and pharmacological properties. Derivatives of 1,4-benzothiazine are widely used as anti-inflammatory (Trapani *et al.*, 1985) and analgesic (Wammack *et al.*, 2002; Yaltirik *et al.*, 2001) drugs. In our previous work we have reported the synthesis of new 1,4-benzothiazine derivatives for biological activities (Zerzouf *et al.*, 2001; Sebbar *et al.*, 2014). The reactivity of propargyl bromide towards 3-oxo-1,4-benzothiazine under phase-transfer catalysis conditions using tetra *n*-butyl ammonium bromide (TBAB) as catalyst and potassium carbonate as base, leads to the formation of the title compound in good yields.

The two fused six-membered rings building the molecule of the title compound are linked to a prop-2-ynyl chain as shown in Fig.1. The six-membered heterocycle of the benzothiazine fragment displays a screw boat conformation as indicated by the puckering amplitude Q = 0.6643 (11) Å, and spherical polar angle θ = 66.56 (9)°, with φ = 330.45 (11)° (Cremer & Pople, 1975). The benzene ring (C1 to C6) makes a dihedral angle of 79.4 (1)° with the mean plan through the prop-2-ynyl chain and the N1 atom (N1C9C10C11).

In the crystal, molecules are linked together by intermolecular C11–H11···O1 interactions forming zigzag chains running along *b* direction (see Fig.2 and Table 1).

### 2. Experimental

To a solution of 3-oxo-1,4-benzothiazine (1.00 g, 6.05 mmol), potassium carbonate (1.52 g,11.3 mmol) and tetra *n*-butyl ammonium bromide (0.12 g, 0.37 mmol) in DMF (25 ml) was added propargyl bromide (0.5 ml, 6.6 mmol). Stirring was continued at room temperature for 24 h. The mixture was filtered and the solvent removed. The residue was extracted with water. The organic compound was chromatographed on a column of silica gel with ethyl acetate-hexane (1/1) as eluent. Orange crystals were isolated when the solvent was allowed to evaporate (yield: 75%, m.p. 493 K).

### 3. Refinement

H atoms were located in a difference map and treated as riding with C—H = 0.93 Å (aromatic, acetylenic) and C—H = 0.97 Å (methylene). All hydrogen atoms were refined isotropically with *U*_iso_(H) = 1.2 *U*_eq_ (aromatic, acetylenic and methylene).

### Crystal data

**Table:**

C_11_H_9_NOS	*F*(000) = 424
*M**_r_* = 203.25	*D*_x_ = 1.376 Mg m^−^^3^
Monoclinic, *P*2_1_/*n*	Mo *K*α radiation, λ = 0.71073 Å
Hall symbol: -P 2yn	Cell parameters from 2532 reflections
*a* = 9.005 (2) Å	θ = 2.7–28.7°
*b* = 10.889 (3) Å	µ = 0.29 mm^−^^1^
*c* = 10.341 (3) Å	*T* = 296 K
β = 104.565 (7)°	Block, colourless
*V* = 981.3 (4) Å^3^	0.39 × 0.34 × 0.28 mm
*Z* = 4	

### Data collection

**Table:**

Bruker X8 APEX diffractometer	2532 independent reflections
Radiation source: fine-focus sealed tube	2242 reflections with *I* > 2σ(*I*)
Graphite monochromator	*R*_int_ = 0.025
φ and ω scans	θ_max_ = 28.7°, θ_min_ = 2.7°
Absorption correction: multi-scan (*SADABS*; Bruker, 2009)	*h* = −12→12
*T*_min_ = 0.692, *T*_max_ = 0.747	*k* = −14→14
9586 measured reflections	*l* = −13→13

### Refinement

**Table:**

Refinement on *F*^2^	Primary atom site location: structure-invariant direct methods
Least-squares matrix: full	Secondary atom site location: difference Fourier map
*R*[*F*^2^ > 2σ(*F*^2^)] = 0.033	Hydrogen site location: inferred from neighbouring sites
*wR*(*F*^2^) = 0.097	H-atom parameters constrained
*S* = 1.05	*w* = 1/[σ^2^(*F*_o_^2^) + (0.0525*P*)^2^ + 0.1971*P*] where *P* = (*F*_o_^2^ + 2*F*_c_^2^)/3
2532 reflections	(Δ/σ)_max_ = 0.001
127 parameters	Δρ_max_ = 0.29 e Å^−^^3^
0 restraints	Δρ_min_ = −0.26 e Å^−^^3^

### Special details

**Table:**

Geometry. All e.s.d.'s (except the e.s.d. in the dihedral angle between two l.s. planes) are estimated using the full covariance matrix. The cell e.s.d.'s are taken into account individually in the estimation of e.s.d.'s in distances, angles and torsion angles; correlations between e.s.d.'s in cell parameters are only used when they are defined by crystal symmetry. An approximate (isotropic) treatment of cell e.s.d.'s is used for estimating e.s.d.'s involving l.s. planes.
Refinement. Refinement of *F*^2^ against all reflections. The weighted *R*-factor *wR* and goodness of fit *S* are based on *F*^2^, conventional *R*-factors *R* are based on *F*, with *F* set to zero for negative *F*^2^. The threshold expression of *F*^2^ > σ(*F*^2^) is used only for calculating *R*-factors(gt) *etc*. and is not relevant to the choice of reflections for refinement. *R*-factors based on *F*^2^ are statistically about twice as large as those based on *F*, and *R*- factors based on all data will be even larger.

### Fractional atomic coordinates and isotropic or equivalent isotropic displacement parameters (Å^2^)

**Table:**

	*x*	*y*	*z*	*U*_iso_*/*U*_eq_	
C1	0.72598 (13)	0.66267 (11)	0.41584 (11)	0.0349 (2)	
C2	0.57459 (15)	0.63028 (14)	0.40891 (14)	0.0468 (3)	
H2	0.5255	0.6635	0.4699	0.056*	
C3	0.49680 (15)	0.54991 (15)	0.31310 (16)	0.0545 (4)	
H3	0.3950	0.5302	0.3081	0.065*	
C4	0.57071 (16)	0.49879 (14)	0.22443 (16)	0.0523 (3)	
H4	0.5185	0.4439	0.1599	0.063*	
C5	0.72189 (15)	0.52826 (12)	0.23035 (13)	0.0432 (3)	
H5	0.7710	0.4922	0.1709	0.052*	
C6	0.80051 (12)	0.61176 (10)	0.32501 (11)	0.0321 (2)	
C7	1.05883 (13)	0.68706 (11)	0.43826 (12)	0.0365 (2)	
C8	1.00619 (14)	0.69227 (13)	0.56496 (12)	0.0400 (3)	
H8A	1.0811	0.7365	0.6327	0.048*	
H8B	0.9981	0.6096	0.5974	0.048*	
C9	1.00187 (16)	0.64258 (13)	0.20101 (12)	0.0430 (3)	
H9A	0.9145	0.6591	0.1264	0.052*	
H9B	1.0779	0.7059	0.2021	0.052*	
C10	1.06719 (14)	0.52290 (14)	0.18089 (12)	0.0430 (3)	
N1	0.95243 (11)	0.64883 (9)	0.32600 (9)	0.0343 (2)	
C11	1.12110 (16)	0.42755 (16)	0.16435 (14)	0.0528 (3)	
H11	1.1637	0.3522	0.1513	0.063*	
O1	1.18902 (11)	0.71561 (11)	0.43539 (11)	0.0552 (3)	
S1	0.82271 (4)	0.76777 (3)	0.53643 (3)	0.04266 (12)	

### Atomic displacement parameters (Å^2^)

**Table:**

	*U*^11^	*U*^22^	*U*^33^	*U*^12^	*U*^13^	*U*^23^
C1	0.0342 (5)	0.0363 (6)	0.0354 (5)	0.0037 (4)	0.0114 (4)	0.0046 (4)
C2	0.0349 (6)	0.0579 (8)	0.0515 (7)	0.0039 (5)	0.0181 (5)	0.0071 (6)
C3	0.0322 (6)	0.0634 (9)	0.0667 (9)	−0.0064 (6)	0.0101 (6)	0.0087 (7)
C4	0.0433 (7)	0.0464 (7)	0.0602 (8)	−0.0078 (6)	−0.0002 (6)	−0.0034 (6)
C5	0.0411 (6)	0.0410 (6)	0.0456 (6)	0.0019 (5)	0.0074 (5)	−0.0055 (5)
C6	0.0299 (5)	0.0317 (5)	0.0350 (5)	0.0015 (4)	0.0086 (4)	0.0038 (4)
C7	0.0342 (5)	0.0343 (6)	0.0421 (6)	−0.0021 (4)	0.0115 (4)	0.0026 (5)
C8	0.0376 (6)	0.0451 (6)	0.0359 (6)	−0.0005 (5)	0.0067 (5)	−0.0010 (5)
C9	0.0458 (7)	0.0512 (7)	0.0373 (6)	0.0020 (5)	0.0202 (5)	0.0070 (5)
C10	0.0364 (6)	0.0614 (8)	0.0346 (6)	0.0008 (5)	0.0155 (5)	−0.0003 (5)
N1	0.0335 (5)	0.0389 (5)	0.0340 (5)	−0.0008 (4)	0.0147 (4)	0.0015 (4)
C11	0.0466 (7)	0.0656 (9)	0.0484 (7)	0.0069 (7)	0.0158 (6)	−0.0085 (7)
O1	0.0370 (5)	0.0686 (7)	0.0619 (6)	−0.0143 (4)	0.0160 (4)	0.0024 (5)
S1	0.0458 (2)	0.04378 (19)	0.04046 (19)	0.00495 (12)	0.01474 (14)	−0.00623 (12)

### Geometric parameters (Å, º)

**Table:**

C1—C2	1.3924 (17)	C7—O1	1.2204 (15)
C1—C6	1.3990 (16)	C7—N1	1.3709 (16)
C1—S1	1.7542 (13)	C7—C8	1.5023 (16)
C2—C3	1.374 (2)	C8—S1	1.8021 (13)
C2—H2	0.9300	C8—H8A	0.9700
C3—C4	1.379 (2)	C8—H8B	0.9700
C3—H3	0.9300	C9—C10	1.4660 (19)
C4—C5	1.385 (2)	C9—N1	1.4707 (14)
C4—H4	0.9300	C9—H9A	0.9700
C5—C6	1.3919 (17)	C9—H9B	0.9700
C5—H5	0.9300	C10—C11	1.177 (2)
C6—N1	1.4239 (14)	C11—H11	0.9300
			
C2—C1—C6	119.60 (12)	N1—C7—C8	116.32 (10)
C2—C1—S1	120.43 (10)	C7—C8—S1	110.60 (9)
C6—C1—S1	119.97 (9)	C7—C8—H8A	109.5
C3—C2—C1	120.88 (13)	S1—C8—H8A	109.5
C3—C2—H2	119.6	C7—C8—H8B	109.5
C1—C2—H2	119.6	S1—C8—H8B	109.5
C2—C3—C4	119.52 (12)	H8A—C8—H8B	108.1
C2—C3—H3	120.2	C10—C9—N1	112.76 (10)
C4—C3—H3	120.2	C10—C9—H9A	109.0
C3—C4—C5	120.71 (13)	N1—C9—H9A	109.0
C3—C4—H4	119.6	C10—C9—H9B	109.0
C5—C4—H4	119.6	N1—C9—H9B	109.0
C4—C5—C6	120.20 (12)	H9A—C9—H9B	107.8
C4—C5—H5	119.9	C11—C10—C9	179.18 (14)
C6—C5—H5	119.9	C7—N1—C6	123.91 (9)
C5—C6—C1	119.07 (11)	C7—N1—C9	117.17 (10)
C5—C6—N1	120.54 (10)	C6—N1—C9	118.89 (10)
C1—C6—N1	120.34 (10)	C10—C11—H11	180.0
O1—C7—N1	121.86 (11)	C1—S1—C8	95.14 (6)
O1—C7—C8	121.83 (12)		

### Hydrogen-bond geometry (Å, º)

**Table:**

*D*—H···*A*	*D*—H	H···*A*	*D*···*A*	*D*—H···*A*
C11—H11···O1^i^	0.93	2.32	3.1937 (19)	157

Symmetry code: (i) −*x*+5/2, *y*−1/2, −*z*+1/2.
